# A validation study of the 4-variable and 8-variable kidney failure risk equation in transplant recipients in the United Kingdom

**DOI:** 10.1186/s12882-021-02259-4

**Published:** 2021-02-09

**Authors:** Ibrahim Ali, Philip A. Kalra

**Affiliations:** 1 Department of Renal Medicine, Salford Royal NHS Foundation Trust, Stott Lane, Salford, M6 8HD UK; 2 Division of Cardiovascular Sciences, University of Manchester, Manchester, M13 9PL UK

**Keywords:** Risk prediction, Kidney failure risk equation, Discrimination, Calibration, Graft failure, Transplant

## Abstract

**Background:**

There is emerging evidence that the 4-variable Kidney Failure Risk Equation (KFRE) can be used for risk prediction of graft failure in transplant recipients. However, geographical validation of the 4-variable KFRE in transplant patients is lacking, as is whether the more extensive 8-variable KFRE improves predictive accuracy. This study aimed to validate the 4- and 8-variable KFRE predictions of the 5-year death-censored risk of graft failure in patients in the United Kingdom.

**Methods:**

A retrospective cohort study involved 415 transplant recipients who had their first renal transplant between 2003 and 2015 and were under follow-up at Salford Royal NHS Foundation Trust. The KFRE risk scores were calculated on variables taken 1-year post-transplant. The area under the receiver operating characteristic curves (AUC) and calibration plots were evaluated to determine discrimination and calibration of the 4- and 8-variable KFREs in the whole cohort as well as in a subgroup analysis of living and deceased donor recipients and in patients with an eGFR< 45 ml/min/1.73m^2^.

**Results:**

There were 16 graft failure events (4%) in the whole cohort. The 4- and 8-variable KFREs showed good discrimination with AUC of 0.743 (95% confidence interval [CI] 0.610–0.876) and 0.751 (95% CI 0.629–0.872) respectively. In patients with an eGFR< 45 ml/min/1.73m^2^, the 8-variable KFRE had good discrimination with an AUC of 0.785 (95% CI 0.558–0.982) but the 4-variable provided excellent discrimination in this group with an AUC of 0.817 (0.646–0.988). Calibration plots however showed poor calibration with risk scores tending to underestimate risk of graft failure in low-risk patients and overestimate risk in high-risk patients, which was seen in the primary and subgroup analyses.

**Conclusions:**

Despite adequate discrimination, the 4- and 8-variable KFREs are imprecise in predicting graft failure in transplant recipients using data 1-year post-transplant. Larger, international studies involving diverse patient populations should be considered to corroborate these findings.

**Supplementary Information:**

The online version contains supplementary material available at 10.1186/s12882-021-02259-4.

## Background

Renal transplantation offers the best long-term outcomes for patients with end-stage renal disease (ESRD) [1, 2]. However, despite advances in treatment to counter short-term transplant complications, many patients still experience late transplant decline and progression to graft failure [3]. In patients with a failing transplant, accurate risk stratification is important to prepare and inform the potential need for renal replacement therapy in a timely manner.

The Kidney Failure Risk Equation (KFRE) is the most extensively validated risk prediction tool for estimating the 2- and 5-year risk of ESRD in patients with chronic kidney disease (CKD) stages 3–5 [4]. The validation of this tool in transplant recipients has been undertaken in 3 studies to date. Two have assessed the 4-variable KFRE, which relies on age, gender, estimated glomerular filtration rate (eGFR) and urine albumin:creatinine ratio (uACR), in North American populations and have shown it can adequately prognosticate graft failure in patients surviving the first-year post-transplant [5, 6]. Most recently, validation of the 4-variable KFRE has also been undertaken in a post-hoc analysis of the Folic Acid for Vascular Outcomes Reduction in Transplantation trial (FAVORIT) and comprised 2889 patients from cohorts in North America and one from Brazil [7]. This analysis also found that the 2- and 5-year risk of graft failure estimated by the 4-variable KFRE can provide adequate risk prediction, but the investigators raised concerns that the risk calculation was imprecise if it was undertaken using data within 2-years post-transplantation.

To date, what is not known is whether the 8-variable KFRE, which in addition to the 4-variable parameters comprises serum calcium, phosphate, bicarbonate and albumin, provides any further improvement to risk prediction in transplant recipients. In addition, further exploration on the accuracy of the KFRE at 1-year post-transplantation is required. In this study, we sought to validate both the 4- and 8-variable KFRE for predicting the 5-year risk of graft failure in transplant recipients. In doing so, we aimed to 1) evaluate the KFREs for the first time, to the best of our knowledge, in a transplant population in the United Kingdom (UK); and 2) provide novel insight of the validity of the 8-variable KFRE in transplant recipients.

## Methods

### Patient population

A single-centre retrospective cohort study was undertaken. All patients aged 18 years or more who had 1) received a renal transplant between 1st January 2003 and 31st July 2015 and were under follow-up at Salford Royal NHS Foundation Trust, and 2) had all measurements available for analysis approximately 1-year post-transplant (and limited up to 18 months post-transplant) were extracted from the hospital’s electronic patient record. Patients who died or reached graft failure (defined as initiating haemodialysis, peritoneal dialysis or receiving another renal transplant) within 1-year of their first transplant were excluded.

### Data variables

All variables for calculating the KFRE measured at least 1-year post-transplant were extracted from the hospital’s electronic record for each patient. These variables enabled the 5-year risk of graft failure to be calculated using the published non-North American 4- and 8-variable KFREs (Additional file 1).

The eGFR was calculated using the Chronic Kidney Disease Epidemiology Collaboration (CKD-EPI) equation. Serum calcium and phosphate, both measured in mmol/L were converted to mg/dL by multiplying values by 4 and 3.1 respectively. Albumin, measured in g/L, was converted to g/dL by dividing values by 10. uACR was estimated from urine protein:creatinine ratio (uPCR) for all patients using a conversion calculation available online, which has been shown to provide good discrimination for use with the KFRE [8]. The uACR units of mg/mmol were switched to mg/g by multiplying values by 8.84. These conversions ensured the unit measurements aligned with the units used in the original development KFRE study [9].

### Study outcome

Death-censored graft failure at 5 years from the point of the 1-year post-transplant measurements was the primary outcome. Outcome data was determined until 31st July 2020 to enable 5-year risk calculations for all patients.

### Subgroup analyses

Given that the KFRE was originally developed to predict risk of ESRD in those with CKD stages 3a-5, a subgroup analysis was performed for patients with an eGFR< 45 ml/min/1.73m^2^, a cut-off value shown to improve prediction performance [6]. The KFRE was also assessed separately in patients with living and deceased donors in the whole cohort and in those with an eGFR< 45 ml/min/1.73m^2^.

### Statistical analysis

For baseline characteristics, continuous data is presented as median (interquartile range) and categorical data as number (percentage). To compare the baseline characteristics between living and deceased donor recipients, *p*-values were calculated by Mann-Whitney test for continuous data and Chi-squared test for categorical data.

To assess the KFRE performance, the discrimination and calibration properties of the 4- and 8-variable KFRE risk scores were evaluated. Discrimination refers to the ability of a model to differentiate high-risk patients from low-risk patients. Receiver-operator characteristic (ROC) curves were created and discrimination was defined by the area under the curve (AUC), along with 95% confidence intervals (CI). An AUC of 1.0 represents perfect discrimination whereas 0.5 means the model’s ability to discriminate cases is no better than chance [10]. Good discrimination is characterised by an AUC of between 0.7–0.8 and excellent discrimination at values > 0.8. Calibration refers to the extent the predicted scores agree with the actual observed data. This was assessed visually by a calibration plot, comparing the predicted risk on the x-axis (split into decile risk groups) with the observed proportion of events in each risk group on the y-axis [10]. Perfect calibration, whereby the predicted probabilities match the observed events, is characterised by an ideal line of 45°.

Statistical analyses were conducted using SPSS (Version 25.0) (IBM SPSS, Chicago, IL), licensed to the University of Manchester. A *p*-value of < 0.05 was considered statistically significant.

### Ethical approval

The study complies with the declaration of Helsinki and was registered with the Research and Innovation department of the Northern Care Alliance NHS Group (Ref: S20HIP57) who approved the methodological protocol as outlined above. As this was a retrospective observational study using measurements routinely collected and using fully anonymised data, the need for individual patient consent was waived by the Research and Innovation review committee, who granted study approval. The study was performed in accordance with the regulations outlined by the review committee.

The reporting of this validation study adheres to recommendations of the TRIPOD (Transparent Reporting of a Multivariable Prediction Model for Individual Prognosis or Diagnosis) statement (Additional file 2) [11].

## Results

### Patient characteristics

**Fig. 1:**
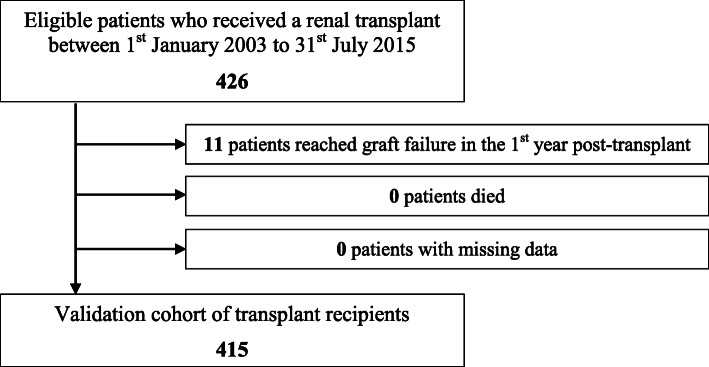
Assembling the study cohort

The inclusion criteria were met by 415 patients (Fig. 1), for whom demographic and laboratory measures are provided in Table 1. The 1-year post-transplant laboratory measurements were taken at a median time-point of 1.07 years (1.03–1.19 years). The median age was 49.8 years (38.9–59.6 years) in the study cohort, which was predominantly Caucasian (88% of patients). The most common underlying disease aetiology was glomerulonephritis. The median eGFR was 54.1 ml/min/1.73m^2^ (41.6–70.5 ml/min/1.73m^2^) and 30% of patients with an eGFR< 45 ml/min/1.73m^2^ comprised part of the subgroup analysis. Of the 415 patients, 97 (24%) were living donor recipients, who were younger in comparison to deceased donor recipients and had a statistically higher level of albumin, although the levels were within normal limits in both groups.

**Table 1:** Characteristics of the study cohort 1-year post-transplant

Variable	All patients (***n*** = 415)	Living donor recipient (***n*** = 97)	Deceased donor recipient (***n*** = 318)	***p***-value^
Age, years	49.8 (38.9–59.6)	40.0 (30.7–53.3)	51.2 (41.5–62.4)	**< 0.001**
Male, *n* (%)	244 (59)	51 (53)	174 (61)	0.155
Caucasian, *n* (%)	352 (88)	97 (100)	311 (98)	0.141
Hypertension, *n* (%)	383 (92)	90 (93)	293 (92)	0.835
Diabetes mellitus, *n* (%)	48 (12)	6 (6)	42 (13)	0.058
**Primary renal disease**				
Glomerulonephritis, *n* (%)	120 (29)	35 (36)	85 (27)	0.075
ADPKD, *n* (%)	55 (13)	14 (14)	41 (13)	0.695
Diabetic nephropathy, *n* (%)	44 (11)	6 (6)	38 (12)	0.106
Hypertensive nephropathy, *n* (%)	21 (5)	5 (5)	16 (5)	0.961
**Laboratory values**				
^*^eGFR, ml/min/1.73m^2^	54.1 (41.6–70.5)	58 (46–75)	53 (40–70)	0.114
eGFR< 45 ml/min/1.73m^2^, *n* (%)	125 (30)	24 (25)	104 (33)	0.137
^†^uACR, mg/g	22.1 (11.5–65.4)	23.9 (12.4–68.1)	21.2 (12.4–64.5)	0.473
^‡^Calcium, mg/dL	9.44 (9.1–9.9)	9.40 (9.08–9.80)	9.48 (9.12–9.96)	0.151
^‡^Phosphate, mg/dL	2.88 (2.43–3.31)	2.88 (2.45–3.38)	2.85 (2.42–3.31)	0.917
Bicarbonate, mEq/L	22.8 (21.2–25.0)	22.6 (21.3–23.8)	23.0 (21.2–25.4)	0.185
^¶^Albumin, g/dL	4.4 (4.2–4.6)	4.5 (4.4–4.7)	4.4 (4.2–4.6)	**0.004**
**Outcome**				
Graft failure within 5 years, *n* (%)	16 (4)	6 (6)	10 (3)	0.173
Time to graft failure, years	3.51 (2.87–4.19)	3.14 (2.86–3.94)	3.62 (3.02–4.14)	0.588

Continuous data are presented as median (interquartile range) and categorical as number (percentage).

^*P*-values calculated by Mann-Whitney test for continuous data and Chi-squared test for categorical data, comparing living with deceased donor recipients. A *p*-value of < 0.05 was considered statistically significant.

^*^eGFR was calculated using the Chronic Kidney Disease Epidemiology Collaboration (CKD-EPI) equation

^†^urine albumin:creatinine ratios were acquired by converting urine protein:creatinine ratios using an online calculator [5] and thereafter switching units from mg/mmol to mg/g by multiplying values by 8.84

^‡^Calcium and phosphate were measured in mmol/L and converted to mg/dL by multiplying values by 4 and 3.1 respectively

^¶^Albumin was measured in g/L and converted to g/dL by dividing by 10

*Abbreviations*: *ADPKD* (autosomal dominant polycystic kidney disease), *eGFR* (estimated glomerular filtration rate)

Within 5-years of follow-up, 16 patients reached the primary outcome of graft failure. A total of 35 patients died prior to graft failure and these patients were censored for the analysis. Additional file 3 compares the baseline characteristics of our cohort with the original KFRE development cohort.

### KFRE performance: discrimination

A summary of the AUCs for the 4- and 8-variable KFREs is shown in Table 2. The 4- and 8-variable KFRE showed good discrimination with AUC values of 0.743 (95% CI 0.610–0.876) and 0.751 (95% CI 0.629–0.872) respectively. In patients with an eGFR< 45 ml/min/1.73m^2^, the 4-varible KFRE had excellent discrimination (AUC 0.817, 95% CI 0.646–0.988) whilst the 8-variable KFRE also demonstrated good discriminatory ability in these patients and had a slightly better AUC of 0.785 (95% CI 0.558–0.982) compared with the 8-variable KFRE in the entire cohort of 0.751 (95% CI 0.629–0.872).

**Table 2:** Summary of discrimination statistics for the 4- and 8-variable KFREs

	Number in group	Graft failure, ***n*** (%)	4-variable KFRE AUC (95% CI)	8-variable KFRE AUC (95% CI)
**All patients**	415	16 (4)	0.743 (0.610–0.876)	0.751 (0.629–0.872)
**Patients with eGFR < 45 ml/min/1.73m**^**2**^	128	9 (7)	0.817 (0.646–0.988)	0.785 (0.558–0.982)
**Deceased donor recipient**	318	10 (3)	0.685 (0.503–0.868)	0.707 (0.544–0.870)
**Living donor recipient**	97	6 (6)	0.846 (0.683–1.000)	0.841 (0.684–0.997)
**Deceased donor recipient eGFR < 45 ml/min/1.73m**^**2**^	104	5 (5)	0.846 (0.663–1.000)	0.800 (0.558–1.000)
**Living donor recipient eGFR < 45 ml/min/1.73m**^**2**^	24	4 (17)	0.787 (0.471–1.000)	0.762 (0.453–1.000)

*Abbreviations* *eGFR* (estimated glomerular filtration rate), *AUC* (area under receiver operator characteristic curve), *CI* (confidence interval)

### KFRE performance: calibration

**Fig. 2:**
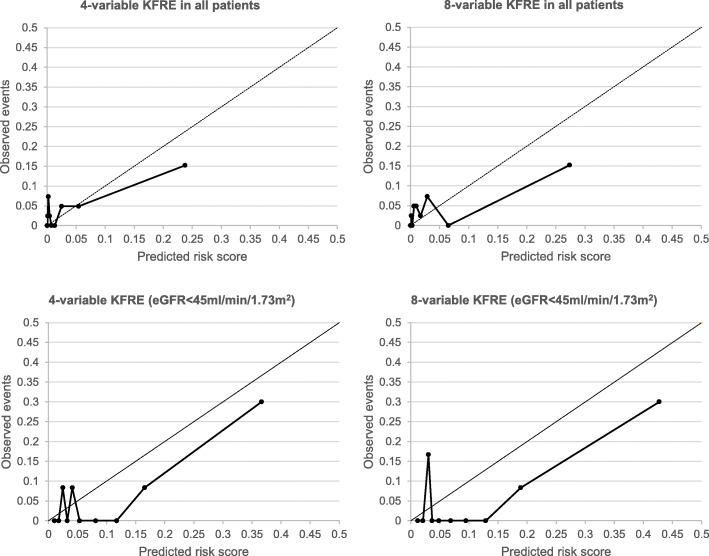
Calibration plots for the 4- and 8-variable KFRE in transplant recipients. Abbreviations: KFRE (Kidney Failure Risk Equation); eGFR (estimated glomerular filtration rate)

The calibration plots shown in Fig. 2 reveal inadequate calibration for the 4- and 8-variable KFRE in the transplant cohort: compared with the perfect calibration slope of 45°, there was a tendency for both the 4- and 8-variable KFRE to underestimate the risk scores at lower risk scores and over-estimate risk in higher risk patients, which was seen within the whole cohort and in those with an eGFR< 45 ml/min/1.73m^2^.

### Donor type subgroup analysis

For the subgroup analysis of living and deceased donor recipients there were 6 outcome events in those with living donors and 10 events in those with deceased donors. The 4- and 8-variable KFRE demonstrated poorer discriminative ability in deceased donor recipients compared to living donor recipients in the whole cohort, but this was improved in those with an eGFR< 45 ml/min/1.73m^2^ (Table 2). Calibration plots, however, revealed that the 4- and 8-varibale KFREs were imperfect in both living and deceased donor groups (Additional file 4) and remained so when further stratified to those with an eGFR< 45 ml/min/1.73m^2^.

## Discussion

This validation study highlights that the 4- and 8-variable KFREs have adequate discriminative ability in predicting the 5-year risk of graft failure in transplant recipients surviving 1-year post-transplant but that they were imprecise with respect to calibration and had a tendency to underestimate risk in low-risk patients and overestimate risk in high-risk patients.

### Comparison with other validation studies using the KFRE

The discriminatory ability of the 4-variable KFRE in our study is in keeping with recently published studies. For instance, Akbari et al. [5] validated the 4-variable KFRE in 877 transplant patients and found the AUC of the 5-year risk of graft failure (based on values taken 1-year post-transplant) to be 0.72 (95% CI 0.69–0.79) in the whole cohort. Similarly, Tangri et al. [6] reported a pooled C-statistic (a measure identical to the AUC for a binary outcome) of 0.73 (95% CI 0.67–0.80) based on data from four different patient cohorts in Canada. These figures align closely with our finding of an AUC of 0.743 (95% CI 0.610–0.876) for the 4-variable KFRE. In addition, Tangri et al. [6] also showed that the pooled C-statistic increased to 0.83 (0.74–0.91) when the KFRE was applied to transplant patients with an eGFR< 45 ml/min/1.73m^2^, and this excellent discrimination is reproduced in our cohort with an AUC of 0.817 (95% CI 0.646–0.988) for the 4-variable KFRE. Similarly, in the recent post-hoc analysis of the FAVORIT trial [7] (in which only patients with eGFR< 60 ml/min/1.73m^2^ were included), Chu et al. found an overall C-statistic of 0.81 (95% CI 0.78–0.84). Whilst they did not undertake a subgroup analysis in patients with an eGFR< 45 ml/min/1.73m^2^, 60.7% of their cohort fell into this eGFR category, highlighting the KFRE demonstrates improved discrimination in patients with more advanced transplant dysfunction.

We show for the first time that the 8-variable KFRE also demonstrated good discrimination in the whole cohort, and although this improved in patients with an eGFR< 45 ml/min/1.73m^2^, it was outperformed by the 4-variable KFRE in this latter group of patients. These differences are likely explained from the incorporation of extra variables in the 8-variable KFRE, such as calcium, phosphate and bicarbonate, which may not offer significant prognostic utility in transplant recipients. In contrast, lower albumin levels at 1-year post-transplant, perhaps reflective of underlying inflammation, have been shown to prognosticate transplant failure [12, 13], and the inclusion of this parameter may explain the slightly better discrimination seen with the 8-variable equation within the whole cohort as compared with the 4-variable KFRE.

However, with respect to calibration, we found the 4- and 8-variable KFRE did not accurately predict observed events. Importantly, miscalibration was demonstrated in the studies by Tangri et al. [3] and Chu et al. [7]. In the latter study, the calibration plots for the 5-year 4-variable KFRE consistently showed an underestimation of risk scores in lower risk patients and overestimation of risk in higher risk patients. This effect was particularly noticeable in patients who had been transplanted for less than 2-years and was the rationale behind the authors’ recommendation to use measurements taken 2-years post-transplant as opposed to 1-year post-transplant when making KFRE calculations. It is also interesting that Akbari et al. [5] found the highest AUC of 0.87 (95% CI 0.83–0.90) when 2-year post-transplant measurements were taken to calculate the KFRE in patients with an eGFR< 60 ml/min/1.73m^2^, compared to 0.76 (95% CI 0.72–0.80) when utilising 1-year post-transplant variables. However, the authors did not report calibration of the KFRE in their study so further work will be necessary to resolve the matter of what time-point post-transplant the KFRE can offer its best predictive performance.

In our subgroup analysis of patients receiving transplants from living and deceased donors, we show the AUC of both the 4- and 8-variable KFREs was higher in living donor recipients compared with deceased donor recipients in the whole cohort. Our findings suggest that donor type affects the ability of the KFRE to risk predict, in contrast to work by Akbari et al. [5] and Chu et al. [7], who both found performance of the KFRE to be similar between living and deceased donor recipients. Phenotypically, the living donor recipients were significantly younger and had higher levels of albumin compared to their deceased donor counterparts, but these variables alone are not sufficient to explain the differential performance of the KFRE. Certainly, living donor recipients are known to have better graft outcomes compared to deceased donor recipients [14] and therefore it is conceivable that transplant-specific differences in our cohort could account for this discrepancy. The weaker discriminative ability in deceased donor recipients was reversed when our analysis focussed on those with an eGFR< 45 ml/min/1.73m^2^ in this group, further highlighting the dependency on eGFR levels on the KFRE risk performance. The favourable discrimination performance, however, was countered by imprecise calibration in this subgroup analysis (Additional file 4), likely attributed to the small number of events in the donor groups.

### Clinical implications

The discrimination performance of the KFRE in our study cohort aligns with other transplant-specific calculators developed to predict graft failure [15], especially for patients with an eGFR< 45 ml/min/1.73m^2^. For instance, Shabir et al. [13] developed a model predicting the 5-year risk of death-censored graft failure and overall graft failure including death, and this comprised sex, ethnicity, and the 1-year post-transplant variables of age, eGFR, uACR, serum albumin and a prior episode of acute rejection. The model validated well in 4 external cohorts with C-statistics for death-censored graft failure ranging from 0.78 to 0.90. A more recent and promising model that has surfaced is the iBox prediction score [16]. This comprises eight functional, histological and immunological variables to predict the 3-, 5- and 7-year risk of graft failure in transplant recipients. A key strength of this model is the extent of external, geographical validation involving 3557 transplant patients across Europe and America and has shown excellent discrimination with a C-statistic of 0.81 (95% CI 0.78–0.84) in Europe and 0.80 (95% CI 0.76–0.84) in America.

Factors such as eGFR and uACR are clearly important predictors of graft failure and thus the KFRE offers an attractive tool for risk prediction given it is an easy-to-use tool, utilises accessible measures, negates the need for histological data and can be incorporated into electronic health systems to provide rapid risk estimation. However, calibration performance cannot be ignored and is often considered the more essential element of a risk prediction tool [10]. Reasons for miscalibration are typically due to differences in the predictor variables between the validation and development cohort as well as differences in the incidence of the outcome event [17]. Our cohort had a low event rate of 16 patients with graft failure and this likely contributed to miscalibration. Nonetheless, the KFRE is clearly limited in precision given that it was originally developed for use in non-transplant patients with CKD stages 3a-5, and hence ignores other factors known to drive transplant deterioration such as human leucocyte antigen mismatching, delayed graft function, episodes of rejection, development of donor specific antibodies, recurrence of primary disease and transplant glomerulopathy [14]. Interestingly, Chu et al. [7] show that the KFRE performance improves in patients 2-years post-transplant, suggesting that early complications such as delayed graft function or rejection predisposing to graft failure may impact the KFRE predictive performance. For now, we would argue that pending further studies on the role that the KFRE offers to transplant risk prediction, clinicians should rely on well-validated transplant-specific algorithms to guide personalised management, such as the iBox tool, which is not limited in performance by eGFR level and can be used for risk evaluation at any point 10 years post-transplant [16].

### Strengths and limitations

We show for the first time the predictive performance of the 8-variable KFRE in transplant recipients, which had been previously postulated as offering better risk prediction than the 4-variable KFRE [5]. We show that whilst the 8-variable KFRE offers good overall discrimination, it is not as strong as the 4-variable KFRE in patients with an eGFR< 45 ml/min/1.73m^2^, likely as a result of lack of predictor power offered by variables such as calcium, phosphate or bicarbonate. This study also delivers for the first time an independent, geographical validation of the KFRE in transplant patients in a UK-based cohort, and corroborates findings previously shown in other cohorts, namely that whilst discrimination is adequate, calibration is imprecise when using 1-year post-transplant variables.

Our study also has important limitations. Firstly, our cohort was small and the event rate low and this likely affected calibration of the KFRE in the whole cohort and subgroup analyses. The sample size in a validation study is determined by the outcome event rate but the adequate number of events to permit analysis remains unclear and there is no universally agreed approach in this regard [18]. What is perhaps relevant for validation studies involved with transplant patients is the recognition that the 5-year rates of graft failure would be expected to be generally low. In the UK, the national average for the 5-year graft failure rate combining both deceased and living donor recipients is approximately 11% based on the 2019 report by the National Health Service Blood and Transport health authority [19]. From the studies in the literature that report the proportion of 5-year events in patients with 1-year post-transplant KFRE calculations, rates are typically less than 10%: Akbari et al. [5] reported 37 events in their single-centre study, which was 4.2% of the whole cohort; Tangri et al. [6] evaluated 4 separate cohorts consisting of 19 (4.1%), 36 (3.8%), 52 (5.2%) and 116 (9.2%) events; and Chu et al. [7] reported a total of 49 (6.0%) events. Thus, whilst our sample size is small, our event rate of 4% is nonetheless similar to previously published studies. Secondly, we were unable to provide the 2-year KFRE risk scores of graft failure as there were no outcome events in this time period. Thirdly, we were required to convert the uPCR to uACR for all the study patients and this may have had an effect on the predicted risk scores. However, many institutions continue to rely on uPCR measurements, and a validated conversion tool now exists as an online calculator [8] to provide a means to obtain reliably converted albuminuria values. Finally, our patient population was derived from a single centre and were largely Caucasian, which limits the generalisability of our findings to other ethnically diverse populations.

## Conclusions

At 1-year post-transplant, the 4- and 8-variable KFREs provide adequate discrimination for predicting graft failure in transplant recipients, especially in those with an eGFR< 45 ml/min/1.73m^2^. However, due to imprecise calibration, their overall predictive performance is limited, and it is likely relevant that these equations do not take transplant-specific variables, such as rejection episodes, into consideration. Additional validation studies of the KFRE using larger, international transplant cohorts would be desirable to corroborate our findings. Future studies should also consider exploring the time-point post-transplant the KFRE offers optimal risk prediction as this would help gauge the potential role the KFRE could play in future transplant care.

## Supplementary Information

**Additional file 1.** The 4- and 8-variable Kidney Failure Risk Equation calculations for the 5-year predicted risk of end-stage renal disease

**Additional file 2.** TRIPOD checklist for reporting of validation studies

**Additional file 3.** Comparison of the study cohort to the KFRE development cohort

**Additional file 4.** Calibration plots for the 4- and 8-variable KFRE for living and deceased donor recipients in the whole cohort and in those with an eGFR< 45 ml/min/1.73m^2^

## Data Availability

The datasets generated and analysed during the current study are not publicly available due to the fact that patients were those managed in a real-world transplant clinic, but they can be made available from the corresponding author on reasonable request.

## Abbreviations

AUC: Area under the curve
CI: Confidence interval
CKD: Chronic kidney disease
eGFR: Estimated glomerular filtration rate
ESRD: End-stage renal disease
KFRE: Kidney Failure Risk Equation
ROC: Receiver operator characteristic
uACR: Urine albumin:creatinine ratio
UK: United Kingdom
uPCR: Urine protein:creatinine ratio
