# Supplementary material for: A validation study of the 4-variable and 8-variable kidney failure risk equation in transplant recipients in the United Kingdom
Source: BMC Nephrol. 2021 Feb 9;22:57. doi: 10.1186/s12882-021-02259-4 (PMC7874608; doi:10.1186/s12882-021-02259-4)
Supplement: Supplementary file 1 — Additional file 1. The 4- and 8-variable Kidney Failure Risk Equation calculations for the 5-year predicted risk of end-stage renal disease [file 12882_2021_2259_MOESM1_ESM.docx]

**Validation of the 4- and 8-variable Kidney Failure Risk Equation in Transplant Recipients in the United Kingdom**

Ibrahim Ali, Philip A. Kalra

**The 4- and 8-variable Kidney Failure Risk Equation calculations for the 5-year predicted risk of end-stage renal disease**

4-variable calibrated non-North American equation:

1 - 0.9365^exp(-0.2201 x (age/10 – 7.036) + 0.2467 x (male – 0.5642) – 0.5567 x (eGFR/5 – 7.222) + 0.4510 x (logACR – 5.137))

8-variable calibrated non-North American equation:

1 - 0.9245 ^ exp(-0.1992 x (age/10 – 7.036) + 0.1602 (male – 0.5642) – 0.4919 x (eGFR/5 – 7.222) + 0.3364 x (logACR – 5.137) – 0.3441 x (albumin – 3.997) + 0.2604 x (phosphate – 3.916) – 0.07354 x (bicarbonate – 25.57) – 0.2228 x (calcium – 9.355))

In the above equations,

Age is the patient’s age in years, at the time of the laboratory measurements.

Male is equal to 1, otherwise 0.

eGFR is the estimated glomerular filtration rate in ml/min/1.73m^2^, calculated using the CKD-EPI equation.

LogACR is the natural logarithm of the urine albumin:creatinine ratio, measured in mg/g.

Albumin is serum albumin measured in mg/dl.

Phosphate is serum phosphate is measured in mg/dl.

Bicarbonate is serum bicarbonate measured in mEq/L

Calcium is serum calcium measured in mg/dl.
