# Supplementary material for: A validation study of the 4-variable and 8-variable kidney failure risk equation in transplant recipients in the United Kingdom
Source: BMC Nephrol. 2021 Feb 9;22:57. doi: 10.1186/s12882-021-02259-4 (PMC7874608; doi:10.1186/s12882-021-02259-4)
Supplement: Supplementary file 3 — Additional file 3. Comparison of the study cohort to the KFRE development cohort [file 12882_2021_2259_MOESM3_ESM.docx]

**Validation of the 4- and 8-variable Kidney Failure Risk Equation in Transplant Recipients in the United Kingdom**

Ibrahim Ali, Philip A. Kalra

**Comparison of the study cohort to the KFRE development cohort**

|  | **Validation cohort in transplant recipients**  **(n = 415)** | **Original KFRE development cohort**  **(n = 3449)** |
| --- | --- | --- |
| **Age, years** | 49 (14) | 70 (14) |
| **Female, *n* (%)** | 171 (41) | 1503 (44) |
| **eGFR, ml/min/1.73m^2^** | 57 (22) | 36 (13) |
| **Serum bicarbonate, mEq/L** | 23 (3) | 26 (4) |
| **Serum calcium, mg/dL** | 9.5 (0.7) | 9.4 (0.6) |
| **Serum phosphate, mg/dL** | 2.9 (0.7) | 4.0 (0.9) |
| **Serum albumin, mg/dL** | 4.4 (0.3) | 4.0 (0.5) |
| **Urine albumin:creatinine ratio, mg/g** | 102 (264) | 93 (378) |
| **Outcome events of graft failure, *n* (%)** | 16 (4) | 386 (11) |

Continuous data is presented as mean (standard deviation) except for urine albumin:creatinine ratio, which is shown as median (interquartile range).

**Abbreviations**: KFRE (Kidney Failure Risk Equation); eGFR (estimated glomerular filtration rate)
