# Supplementary material for: A validation study of the 4-variable and 8-variable kidney failure risk equation in transplant recipients in the United Kingdom
Source: BMC Nephrol. 2021 Feb 9;22:57. doi: 10.1186/s12882-021-02259-4 (PMC7874608; doi:10.1186/s12882-021-02259-4)

**Validation of the 4- and 8-variable Kidney Failure Risk Equation in Transplant Recipients in the United Kingdom**

Ibrahim Ali, Philip A. Kalra

**Calibration plots for the 4- and 8-variable KFRE in living and deceased donor recipients in the whole cohort and in those with an eGFR<45ml/min/1.73m^2^**

**Living donor recipients**


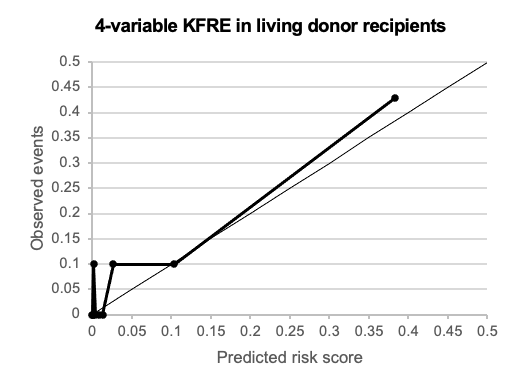

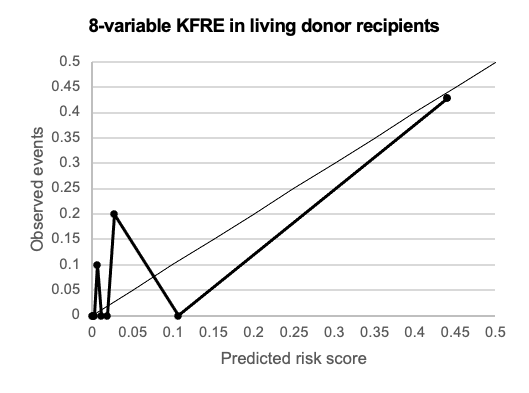


**Deceased donor recipients**


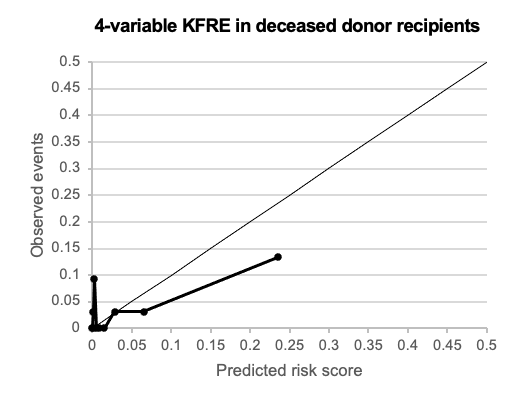

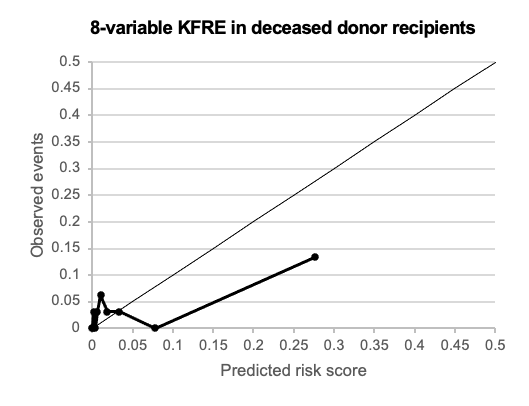


**Living donor recipients (eGFR<45ml/min/1.73m^2^)**


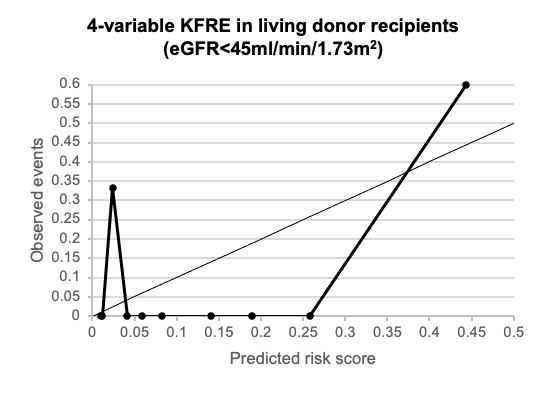

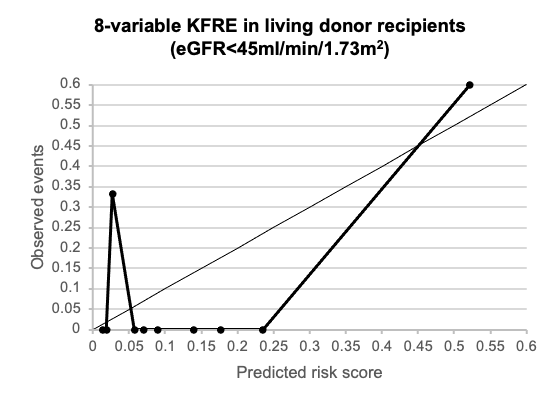


**Deceased donor recipients (eGFR<45ml/min/1.73m^2^)**


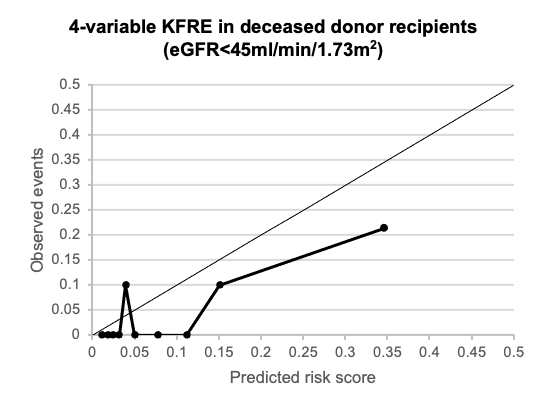

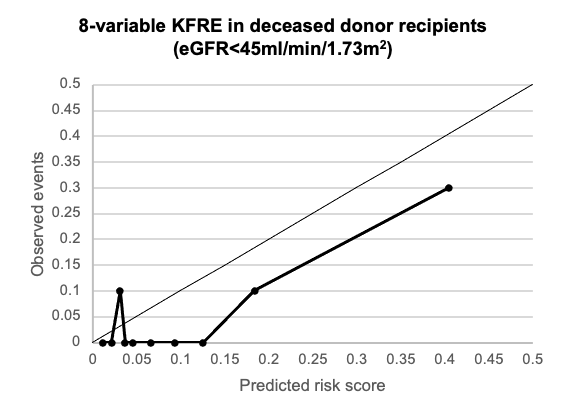

Supplement: Supplementary file 4 — Additional file 4. Calibration plots for the 4- and 8-variable KFRE for living and deceased donor recipients in the whole cohort and in those with an eGFR< 45 ml/min/1.73m2 [file 12882_2021_2259_MOESM4_ESM.docx]
